# Nutrition Knowledge as a Driver of Adherence to the Mediterranean Diet in Italy

**DOI:** 10.3389/fnut.2022.804865

**Published:** 2022-03-21

**Authors:** Vittoria Aureli, Laura Rossi

**Affiliations:** CREA Council for Agricultural Research and Economics-Research Centre for Food and Nutrition, Rome, Italy

**Keywords:** adherence to Mediterranean diet, nutrition knowledge, socioeconomic characteristics, consumers, Italy

## Abstract

**Background:**

An optimal Nutrition knowledge (NK) among the population could result in greater Adherence to the Mediterranean Diet (AMD), a recognized dietary pattern capable of preventing chronic food-related diseases. This study aimed to evaluate the association between NK and AMD in Italy.

**Methods:**

A national representative sample of 2,869 adults took part in an assessment that was carried out through a self-administrated questionnaire including sections relating to NK and AMD. AMD was evaluated following the PREDIMED PLUS methodology. Descriptive statistics were provided, and ordinal measures of NK score and AMD were calculated based on quartiles of the quantitative scores. A contingency analysis was performed to check associations between variables.

**Results:**

In Italy, the average NK score was 50 ± 13.3, equivalent to 56.8% of correct answers. The average value of AMD was 6.8, corresponding to 40% of the maximum score with 31.4% of the population demonstrating low AMD, 31.3% in the lower-middle range, 24% in the medium-high range, and only 13.3% reporting a high AMD. A significant association between NK and AMD was found; respondents who reported the lowest AMD corresponded to those with the lowest NK (36.7%; *p* < 0.05) and similarly, those with the highest level of adherence to MD also achieved the highest NK scores (41.7%; *p* < 0.05).

**Conclusion:**

This study showed that AMD in Italy is generally low, and the strong association between NK and AMD demonstrated that there is a clear connection between a healthy dietary pattern and the nutrition literacy of the population. Those with the highest AMD corresponded to the highest NK and, conversely, those with the lowest AMD displayed the lowest NK. The study also highlighted that socioeconomic aspects were strong determinants of both AMD and NK.

## Introduction

Underweight and obesity are widespread global problems, with more than 820 million people in the world who do not have enough food to cover their daily caloric needs, and a further significant segment of the population who have an excess of food (1). Recent data reported an overall world prevalence of obesity of 13.1 (2), a condition which is strongly linked to the development of Chronic Food-related Diseases (CFD-cardiovascular diseases, diabetes, cancer). The evidence of the close interconnection between CFD and being overweight is a key focus of preventive nutrition programs which have the scope of increasing people’s awareness of the preventive role of healthy food choices. In fact, according to Taylor et al. (3), nutrition literacy is a key factor in the prediction of adherence to healthy/unhealthy dietary patterns. Food policy also relies heavily on the knowledge of consumers, since the information available to the public through education and dietary guidelines influences people’s behavior, enabling them to make better choices (4). Nutrition Knowledge (NK) is considered as one of the factors affecting food habits and food consumption patterns (5). However, NK is strongly influenced by environmental variables such as the behavior of the family (6, 7), school (8), as well as external inputs such as television advertisements (9, 10) or modern eating habits. In addition, socio-economic and educational levels have an important impact, especially in the youngest age group of the population (11).

The Mediterranean Diet (MD) is largely recognized as a nutritional pattern capable of preventing serious pathologies such as cardiovascular diseases (12), diabetes (13), chronic kidney disease (14), and reducing causes of mortality (15). However, in recent years, the progressive abandoning of MD principles has been observed especially in the populations of the Mediterranean basin area. As reported by Vilarnau et al. (16), the Mediterranean Adequacy Index (MAI) declined significantly in the period between 1961–1965 and 2000–2003 globally; a stabilization was observed in the period between 2004 and 2011, with a positive trend of MAI in 16 countries not located in the Mediterranean region (North and Central Europe and North America). These changes were observed also in Italy with the same South-North gradient regarding the abandonment of traditional dietary patterns. According to a national study, Southern Italian regions still showed better adherence to the MD than the Northern regions (17); however, since 1985–1986, in South Italy a progressive decline in the adherence to the MD in all age groups has been seen, with the highest rate amongst the younger age groups (18). Age is in fact an important determinant in adherence to the MD, with the elderly keener to choose traditional meals whilst young people are more likely to select more globalized and western style foods (19). The Western-style diet is characterized by the use of large quantities of ultra-processed and high-calorie foods, typically rich in proteins, saturated fatty acids, and sugars, and by a progressive reduction of plant-based foods (cereals, legumes, fruits, and vegetables). Daily portion sizes also see an increase of 30–40% despite there being a reduction in daily energy expenditure, mainly related to a more sedentary behavior and typology of work (20, 21). The protective value of the MD for health is a well-established concept, however, in recent years its positive impact on the environment has also been highlighted. The large proportion of plant-origin products with small quantities of animal source foods means that this food pattern has a lower ecological footprint than other dietary patterns (22–24). Thus, the Mediterranean Dietary Pattern is potentially the best evidence-based, healthy, and sustainable diet (21).

In this context, it becomes essential to educate consumers to enable them to make more informed and healthy food choices both to promote health and to protect the environment. An optimal NK is linked to greater adherence to the Mediterranean model and a lower prevalence of obesity (25, 26), as well as to a reduction in body mass index (BMI), waist circumference, and fat mass (27, 28). However, public health nutrition recommendations promoting the benefits of daily consumption of fruits and vegetables, the concept of a balanced diet, and the importance of reducing the intake of saturated fats are still not well known by large groups of the population in Italy (29). Hence, the collection of information regarding the assessment of the NK of the population and selected groups is important to permit targeted interventions and duly shaped corrective measures to be put in place.

The main purpose of this study was to measure the NK and the Adherence to Mediterranean Diet (AMD) in the Italian adult population and to evaluate how these factors are correlated. The hypothesis underlying the work is that a high level of NK would correspond to better nutritional outcomes in terms of food consumption and health-promoting dietary habits. More specifically, the study aimed to evaluate the territorial variability of NK and AMD in terms of the socio-economic characteristics and cultural diversity of Italian regions. Finally, the data collected in the present work can be used for monitoring and benchmark purposes, updating information on the AMD of the Italian population and the evaluation at national level of NK that in Italy in the past has been measured only in selected population samples.

## Materials and Methods

### Design of the Study

A cross-sectional survey was carried out on a sample of 2,869 respondents, representative of the Italian adult population (age > 18 years) in the period between 26 June and 10 July 2020. This period was selected in consideration of the fact that the social restrictions related to the COVID-19 pandemic in Italy were attenuated from the 18 May 2020 and then further reduced after the 3rd of June 2020 with the reopening of all activities and free circulation between regions. The fieldwork was carried out by a specialized market research agency, SWG Italy^®^. The sample was stratified by area of residence, age, gender, education, and family size to represent the Italian census figures. The data were collected mainly through online interviews using the CAWI (Computer Assisted Web Interviewing) technique on a sample of adults residing in Italy (*n* = 2619), extracted from a Panel that includes over 60,000 individuals profiled according to the main socio-demographic variables. In order to be included in the SWG consumer panel, respondents were required to sign a privacy policy and consent form in advance for the collection and processing of personal data in accordance with the Italian Data Protection Law (Legislative Decree 101/2018) and the European Commission General Data Protection Regulation (679/2016). The final sample size was reached by adding a group of 250 people who did not use the Internet and were not familiar with communication networks. This group was interviewed through the CAPI (Computer Assisted Personal Interview) technique directly by the operators. This was made possible thanks to the fact that SWG provided a group of interviewers specialized in direct interviews and trained for this activity. The questionnaire was completed by the respondents themselves. This study was conducted according to the guidelines of the Declaration of Helsinki (30), and all procedures involving research study participants were approved and are in line with the SWG Code of Conduct (31). The assessment did not involve any invasive procedure or induce any change in dietary patterns. Before starting the data collection, participants were informed about the objective of the research and the consequent statistical analysis, and about the intention to publish the results in scientific papers. Participation in the study was fully voluntary and anonymous and subjects could withdraw from the study at any time and for any reason. For this reason, according to national regulations, the study did not require approval by Ethics Committee.

### Assessment Tool

The measurements made were modeled according to the objective of the present study. An multi-section questionnaire was administrated including an initial part covering sociodemographic information (gender, age, region of residence, education, income) and self-reported weight and height. The key elements of the assessment tool consisted of two main modules: (i) the Italian Nutrition Knowledge questionnaire and (ii) the Adherence to Mediterranean Diet questionnaire. These two modules built on the work carried out in previous studies (32, 33) in which the methodologies of data collection were tested and adapted to the Italian context.

#### The Italian Nutrition Knowledge Questionnaire

The General Nutrition Knowledge Questionnaire (GNKQ) is one of the most widely used and validated scientific tools for assessing NK in the adult population; it was developed in the 1990s (34) and subsequently adapted for use in different settings (35, 36). Kliemann et al. (37) updated the questionnaire conceived by Parmenter and Wandle (34) to include the most recent nutritional recommendations (GNKQ-R). For the present study, we adapted the GNKQ-R to the Italian nutritional context, taking into consideration the recommendations of the latest Italian Dietary Guidelines (38). This adaptation and validation included work carried out in Scalvedi et al. (32) in which an Italian version of the NK questionnaire (I-NK) was developed and used for a cross-sectional assessment.

Details of the changes and the philosophy of I-NK development are fully explained in Scalvedi et al. (32). The I-NK questionnaire used in this work is reported in Supplementary Material A. In summary, it was composed of four sections exploring different aspects of NK: (NK1) Experts’ recommendations (9 questions); (NK2) Food composition (10 questions); (NK3) Food choices and nutrition labels (11 questions); (NK4) Diet-disease associations (16 questions).

The I-NK consisted of closed-ended, multiple-choice, and yes/no questions. The scoring system applied was: +1 point for each correct answer, 0 points for “I don’t know” or wrong answers.

#### Adherence to Mediterranean Diet Questionnaire

The PREDIMED study (PREvención con DIeta MEDiterránea) largely confirmed the protective role of the Mediterranean Diet (MD) against cardiovascular diseases (stroke, myocardial infarction, and cardiovascular death) (39, 40). An important instrument developed in the framework of this activity was the PREDIMED PLUS questionnaire aimed at assessing adherence to the MD (41). The questionnaire has been used widely in a number of EU countries (42–44). In a recent article focused on the evaluation of dietary changes during the COVID-19 pandemic, we adapted and tested the PREDIMED PLUS in the Italian context (33). The questionnaire asked about the frequency of consumption of traditional Mediterranean food (daily or weekly) by defining the portion size, or asking about the use of food with “yes or no” options, or identifying preferences between different food options. Concerning the original PREDIMED PLUS questionnaire (41), although this is not validated on the Italian population, the portion sizes were modified according to those defined by Italian nutritional recommendations, using the country-specific Dietary Guidelines (45). No other changes were introduced in terms of food groups, scoring, and the calculation of adherence to the Mediterranean Diet (AMD). The final version of the PREDIMED PLUS used in Italy can be found in Grant et al. (33).

Based on the PREDIMED PLUS scoring, AMD was classified into four categories: low (score 0–6), low to moderate (score 7–8), moderate to high (score 9–10), and high (score 11–17) (31).

### Data Analysis

Descriptive statistics were performed to describe the main features of the phenomena being studied. Ordinal measures of NK score and AMD were built based on quartiles of the quantitative scores. A contingency analysis was performed to check associations between variables. More specifically, double-entry tables were processed, and the Chi-squared test of independence was applied along with *post-hoc* tests to check pairwise comparisons with Bonferroni corrections of the *p*-values. The statistical analysis was performed using the IBM SPSS Statistics, version 25.

## Results

### The Characteristics of the Sample

Table 1 shows the socio-demographic characteristics of the sample examined that are in line with the Italian socio-demographic composition (46). This is consistent with the sampling procedure and with the weighting of the data carried out in order to build a sample that was as representative as possible of the Italian population. Calculation of Body Mass Index (BMI) from self-reported weight and height, showed that almost half of the sample (47%) had values in the range of normality, while 35% of respondents were overweight and 15% of the population were classified as obese. Being underweight is an uncommon condition in Italy (3%) (Table 1).

**TABLE 1:** Population sociodemographic information and Body Mass Index (BMI).

Gender	(%)			Family size	(%)
Female	52			1 person	13
Male	48			2 people	34
				3 people	25
**Age**				4 people	22
18–24 years	8			5 or more people	7
25–34 years	13				
35–44 years	16			**Household income**	
45–54 years	19			Up to 18.000 euro	21
55–64 years	17			Between 18,001 and 27,000 euros	24
>64 years	27			Between 27,001 and 36,000 euros	19
				Between 36,001 and 54,000 euros	15
**Education level**				Between 54,001 and 72,000 euros	5
Low	49			Between 72,001 and more	4
Medium	36			Prefer not to answer	13
High	15				
				**Origin’s Area (macro-regions and regions)**	
**Job**				Alpine Area (Piemonte, Liguria, Valle D’Aosta)	10
Self-employed	14			Lombardia	17
Employees	35	Office work	75	Northwest Area (Trentino Alto Adige, Friuli Venezia Giulia)	4
		Highly specialized work	25	Veneto	8
Student	6			Emilia-Romagna	7
Seeking first job/unemployed	9			Apennine Area (Toscana, Umbria)	8
Householder	12			Adriatic Area (Marche, Abruzzo)	5
Retired from work/pensioner	23			Lazio	10
				Southwest Area (Puglia, Molise)	7
**Body mass index**				Campania	9
Underweight	3			Southeast Area (Calabria, Basilicata)	4
Normal weight	47			Islands (Sicilia, Sardegna)	11
Overweight	35				
Obese	15				

### The Nutrition Knowledge Assessment

The average overall NK score was 50 ± 13.3, the equivalent of 56.8% correct answers. Diet-disease association (NK4) was the section where the best results were achieved (60.2%), while food composition (NK2) had the lowest score (53.5%) (Table 2).

**TABLE 2:** Nutrition knowledge assessment in Italy.

	Total NK	NK1- Experts’ recommendations	NK2- Food composition	NK3- Food choices and nutrition label	NK4 -Diet-disease associations
Mean (a)	50.0	10.7	19.3	7.4	12.6
SD	13.3	3.1	6.0	2.6	3.9
Theoretical max (b)	88	18	36	13	21
NK rate (a)/(b)	56.8%	59.7%	53.5%	56.6%	60.2%

Socio-demographic aspects were found to be associated with overall NK scores, expressed in quartiles (Supplementary Material B: Table 3). The most significant NK determining factors were gender (25.1% female vs. 18.9% male, *p* < 0.001) and age, with respondents 45–54 years old having significantly higher NK (28%, *p* < 0.03) than other age groups. A statistically significant NK gradient was observed in connection with educational level (34.0% high vs. 26.4% medium vs. 15.4% low, *p* < 0.01). The highest NK scores (36.7%, *p* < 0.05) were found in the high-income class group (54,000–74,000€). Geographical NK differences were found with North-Central regions showing higher values of NK (Lazio, 29.5% and Emilia Romagna, 27.3%) than Southern regions (Molise 3.3%, *p* < 0.03). The degree of urbanization was strongly associated with NK since approximately one- quarter (39.2%) of respondents living in low population density municipalities showed the lowest level of NK (*p* < 0.001).

**TABLE 3:** Association between nutrition knowledge and Adherence to Mediterranean Diet in Italy *p < 0.05.

Nutrition knowledge quartiles	Low (0 < 6)		Low-medium (6–7)		Medium-high (8–9)		High (10–17)		Total	
	*n*	%	*n*	%	*n*	%	*n*	%	*n*	%
**Adherence to Mediterranean Diet categories**										
Low (0–42)	331	36.7*	245	27.3	126	18.3	26	6.7	728	25.4
Low-Medium (43–52)	257	28.6*	244	27.1	182	26.4	77	20.2	760	26.5
Medium-High (53–60)	179	19.9	238	26.5	209	30.3	120	31.4*	746	26.0
High (>60)	133	14.8	171	19.0	172	25.0	159	41.7*	635	22.1

#### NK1-Expert Recommendations

The NK1 assessment showed that the Expert’s recommendations section gained 60% of correct answers with an average score of 10.75 out of 18 as the theoretical maximum. In the sub-sample of non-computerized respondents, the proportion of correct answers went down to 57% (not significant). In terms of sociodemographic characteristics, respondents residing in central Italian regions (the Apennine area and Lazio), together with those with a high level of education, showed higher NK1 scores than the average of the sample. On the other hand, respondents aged 33–44 years, living in the Veneto region (North Italy), with low education level and large families, obtained a lower NK1 score than the average for the general population (52–56%). Respondents with normal values of BMI obtained 61% of correct answers, while this percentage was 59% in the overweight and obese respondents (data not shown).

**FIGURE 1:**
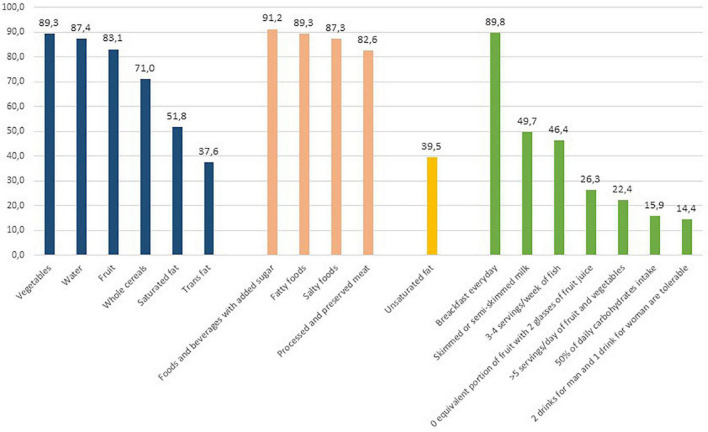
Percentages of correct answers of the NK1-expert’s recommendations section.

Figure 1 reports the percentage of correct answers of the NK1 section. The recommendation related to the increase in consumption of whole-grain cereals was the least well-known (71%) in Italy. A general lack of knowledge was found on the recommendations related to fat consumption, with almost half of the population reporting that they did not know the importance of reducing saturated fat intake, and only 39.5% answering that unsaturated fats are part of a healthy diet. Other questions in the NK1 section were related to the recommendation to eat 5 portions a day of fruit and vegetables, which was known by 22.4% of the population. Only 26.3% of respondents were aware that two glasses of fruit juice did not correspond to a portion of fruit. Almost half of the respondents correctly reported the recommended type of milk (semi-skimmed) (49.7%) and the correct frequency of consumption of fish (3–4 times per week) (46.4%). The daily base consumption of carbohydrates as an element of a healthy diet was reported by only 15.9% of respondents.

#### NK2-Food Composition

In the NK2 section, an overall average score of 19.27 out of 36 as the theoretical maximum was obtained, corresponding to 54% of correct answers; lower scores (18.80/36 corresponding to 52% of correct answers) were found in non-computerized respondents (not significant). In terms of sociodemographic characteristics, the highest NK2 scores were seen for respondents aged between 55 and 64 years old, those residing in the north (Emilia-Romagna) and central regions (Lazio), with a high level of education and, and young students. In contrast, subjects aged between 35 and 44 years, residing in the eastern regions (Adriatic area), with large family sizes (more than 5 components) scored lower than the overall average. Stratification for BMI showed a slightly higher NK2 score for those of normal weight compared to overweight and obese respondents (data not shown).

**FIGURE 2:**
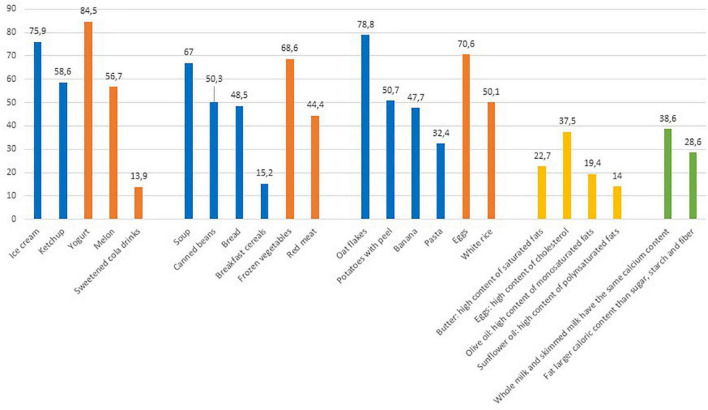
Percentages of correct answers to selected questions of NK2-food composition section. The reported items were those with the general lowest scoring.

In Figure 2 the correct answers to the lowest scoring questions of the NK2 section are reported. The content of sugar and salt of the different foods was correctly identified by almost half of the population, with a few exceptions; a limited proportion (13.9%) of respondents considered sweetened cola drinks low-sugar foods and only a minority of respondents (15.2%) knew that breakfast cereals were a hidden source of salt. Pasta and banana were recognized as a source of fiber by 47.7 and 32.4% of the population, respectively. In terms of section NK1, the composition of fats was the least familiar for Italian consumers, resulting in the lowest percentages of correct answers and the highest selection of the “I don’t know” option. Other misconceptions regarded milk, with about one-third of the population mistakenly considering whole milk as richer in calcium than skimmed milk (34%) and sugars thought to be higher in calories than fats by 36% of respondents.

#### NK3-Food Choices and Nutrition Labels

In the NK3 section on food choices and nutrition labels respondents achieved an average score of 7.36 out of 13 as the theoretical maximum, corresponding to 57% of correct answers; the lowest scores (6.63/13, corresponding to 51% of correct answers) were found in the non-computerized group of respondents (not significant). The results of the NK3 section showed that the most informed consumers were those residing in Lazio (60%), subjects with high education (64%), students (61%), employees with a high specialization (60%), and subjects with a high family income (60–61%). The subsample that scored lower than the average were subjects aged between 34 and 55 years (53%), residents in the East Area regions (50%), and living in the largest families (51%). Furthermore, BMI did not influence NK3 scoring (data not shown).

**FIGURE 3:**
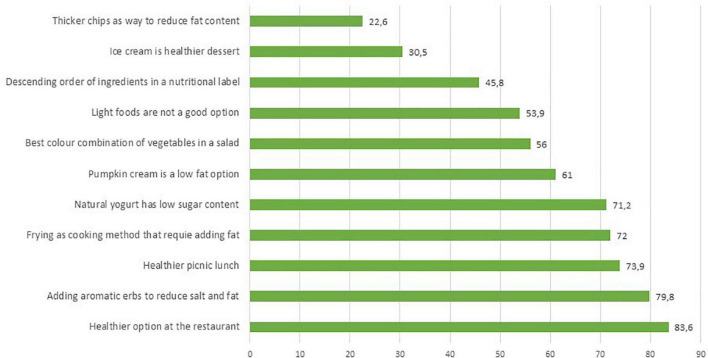
Percentages of correct answers of the NK3–food choices section.

This set of questions provided a list of options allowing respondents to select the healthy food or the most balanced meal, or the healthy cooking method (Supplementary Material A). Only one-third (30.5%) of respondents selected ice cream as the healthiest dessert with a quota of 10.7% that selected the “I don’t know” option. Less than half of the respondents (45.5%) were able to answer the question on the best way to cut potatoes to limit fat content during frying (Figure 3).

The NK3 section included two questions related to the ability to read and understand nutritional labels. A large percentage (67.6%) of the population was able to identify the product with the highest energy content between two labels proposed as models, but only 17.7% were able to recognize the different sources of sugars from the list of ingredients. The population groups with the worse scores were adults over 55 years old, residing in central Italy, with a family of up to 2 people who did not use the internet (data not shown).

#### NK4-Diet-Disease Associations

Section NK4 on the relationship between nutrition and health was the group of questions for which respondents achieved the best scores–12.64 out of 21 as the theoretical maximum, corresponding to 60% of correct answers. As in the other sections lower scores for non-computerized subjects (12.00/21 corresponding to 57%) was observed (not significant). The most informed subjects on the NK4 section were those aged between 55 and 64 (64%) and with an income of over 72 K (65%); on the other hand, the respondents who achieved the lowest scores were those under 44 years old (56–57%), residing in the Eastern regions (North and Adriatic Area) (57%), families with more than 5 components (55%) and with a low income (57%) (data not shown).

**FIGURE 4:**
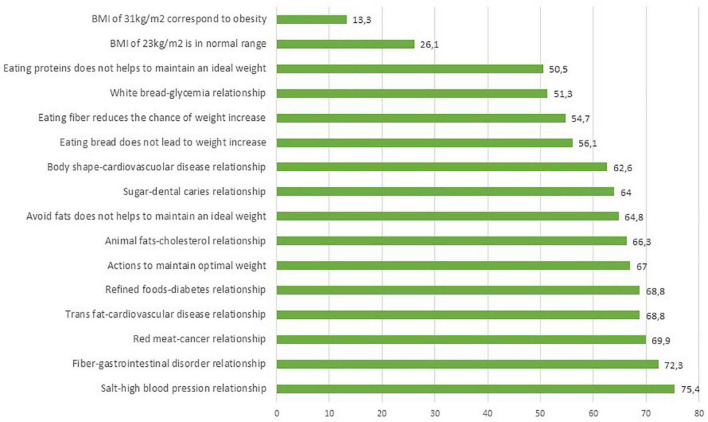
Percentages of correct answers of the NK4–diet-diseases associations section.

In section NK4, 14 questions out of 16 were answered correctly by 50% of respondents (see Figure 4). Interestingly, just over half of the population (53%) selected the “I don’t know” option for the two questions regarding BMI. Respondents were therefore unaware of the meaning of BMI and its use in determining ponderal index. The percentage of those who correctly identified normal weight was only 26.1% and even lower (13.3%) for obesity.

### Adherence to Mediterranean Diet

AMD in Italy was, on average, 6.8, corresponding to 40% of the maximum score. According to AMD categorization, 31.4% of the population had low AMD, 31.3% were in the lower-middle range, 24% in the medium-high range, and only 13.3% with high AMD.

Sociodemographic characteristics were found to be significantly associated with AMD (Supplementary Material C: Table 4). Females showed higher AMD than males (16.6 vs. 9.7%, *p* < 0.001). The youngest respondents showed lower adherence than elderly (low AMD: 39.9% for age 18–24 vs. 27.6% for age 55–64, and 26.4% for age > 64, *p* < 0.01). It should be pointed out that among the elderly (age > 64), one respondent in five showed the highest level of AMD (*p* < 0.05). North-eastern regions and Campania (a region in the south) showed the lowest AMD level (respectively, 45.4 and 44.2%), while the regions of Emilia-Romagna (north) and Lazio (center), as well as the islands (Sicily and Sardinia), showed the highest AMD levels (respectively, 17.2, 16.2, and 17.7%). Living in urbanized areas is associated with a high level of ADM (14.3% high urbanization vs. 8.3% low urbanization, *p* < 0.01). Education is strongly associated with AMD: a high proportion of those with low AMD (35%) was found in the population group with a low level of education, while respondents with a high level of education had a significantly higher AMD rate (14.1% high education vs. 11.4% low education, *p* < 0.01). In terms of family size, living in large families is associated with low AMD compared to respondents living alone or in a family with 2 components. AMD was found to be higher in those of normal-weight (41%) than in overweight respondents (39%) (data not shown).

**FIGURE 5:**
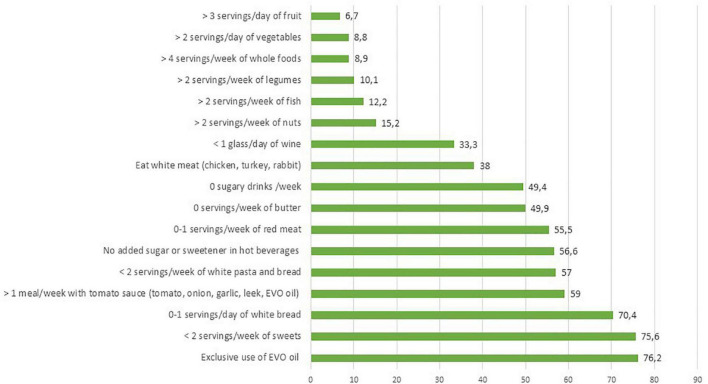
Prevalence of answers in line with adherence to the Mediterranean diet.

In Figure 5 the prevalence of answers in line with the MD according to the PREDIMED PLUS are reported. In Italy, dietary habits coherent with MD principles were observed frequently especially for olive oil, tomato sauces, and white meat consumption. The habit of not adding sugar to drinks was reported by 56.6% of the population, and consumption of sweets and pastries was not reported frequently. One-third of consumers reported an intake of more than 1 glass of wine per day. Eating habits that vary from the Mediterranean Diet recommendations were reported for consumption of nuts, fish, legumes, whole cereals, vegetables, and fruits, corresponding to the recommendations for a limited proportion of the population (ranging from 15.2 to 6.7%). In addition, nearly half of the sample consumed red meat more than once a week and did not follow the recommendations for consumption of whole-grain cereals.

Women and underweight respondents reported the highest frequencies of consumption of vegetables; fruit is consumed more frequently among respondents living on the islands and among those who have normal weight. The consumption of legumes was highest among students and consumers with a medium income (27,001–36,000€). Fish consumption is lowest in large families (>3 people) and highest in those with a medium-high income. Nuts were consumed in lower quantities by males and by respondents with a low level of education and in the highest quantities by singles and by people with high income (54,001–72,000€). Consumption of whole cereals was most common in the normal weight respondents and among students. The meat consumption pattern in Italy showed interesting results. Almost half of the respondents (53%) declared that they consumed all types of meat (red and white), while 38.0% consume mainly white meat. All types of meat are consumed mainly by men, by those who are obese, in large families (4 people), and in families with an income >52,001€. The highest consumption of red meat was found in people who do not use the Internet, while frequent users of the Internet (more than 12 h a day) declared that they did not consume meat at all (data not shown).

### Association Between Nutrition Knowledge and Adherence to the Mediterranean Diet

To test the study hypothesis that a high level of NK would correspond to a better food consumption pattern, the association between NK and AMD was assessed and the results were reported in Table 3. To assess AMD in Italy the resulting scores were divided into low (scores < 6), low-medium (scores 6–7), medium-high (scores 8–9), and high (scores 10–17). Quartiles were identified to define the NK levels of Italians: low (scores 0–42), low-medium (scores 43–52), medium-high (scores 53–60), and high (scores > 60). The results demonstrated that in Italy there was a significant association between NK and AMD. Specifically, the chi-squared test applied on the contingency table reporting the joint frequency distribution of the two ordinal variables, provided significant results (Chi-squared = 236, 13, *p* < 0.05). The extreme classes of NK and AMD categories are particularly correlated, and there is a clear gradient as far as the values in between are concerned. The lowest AMD corresponds to the lowest NK (36.7%; *p* < 0.05);conversely, as shown in the last row of Table 3, the higher the level of adherence to MD, the higher the NK score (41.7%; *p* < 0.05).

## Discussion

This study measured NK and AMD in Italy and provided interesting results that could be useful both as a benchmark and for monitoring purposes. In addition, the present data could be used as a reference for other studies aiming to collect data on the same indicators in specific settings that could be then compared with national-level data. The added value of this assessment is related to the fact that, to date, and to the best of our knowledge, this is the first survey carried out in Italy measuring NK and AMD at the household level on a representative national sample. Other measurements of the same indicators were previously carried out on population groups (21, 47–49) and in selected settings (50, 51). This study provided national measurements that, in line with the declared objectives, assess the levels of NK in Italy and update the level of AMD among the population.

The most significant result of this study is the identification of a strong correlation between NK and AMD in Italy. The survey carried out demonstrated that the lowest AMD corresponds to the lowest NK and the higher the level of adherence to MD, the higher the NK score. In Italy, a study done by Bonaccio et al. (24) was the first to measure the association of NK with AMD, which was later confirmed by Scalvedi et al. (32) which showed that individuals with dietary habits that adhere closely to nutritional recommendations also had a high NK score and subjects with unhealthy dietary habits showed low NK scores. The association between NK and the quality of dietary intake is an open question, with most studies reporting a significant and positive association between NK and some aspects of dietary intake, few reporting negative associations, and approximately one-third that failed to observe any association (52). However, the relationship between NK and AMD seems to be more consolidated than the association of NK with dietary intake. According to Neshatbini Tehrani et al. (25), a high NK score is significantly associated with a high AMD score in Iranian female adolescents, with girls in the highest tertile of the NK score having a higher adherence to MD compared with those in the lowest tertile.

Our data showed a national level of NK correct answers of 56.8%. Comparison of these results could be carried out with the data provided in Scalvedi et al. (32) that in a sample taken Rome and surroundings areas reported 46% of correct NK answers, and with data from Bonaccio et al. (24) that in a sample of south Italy region (Molise) reported 40% of correct answers. The higher NK level of the present data respect to other Italian studies could be explained by analyzing the various different socio-economic contexts. Covering the whole Italian population, the effect of rural areas, that are highly represented both in the Molise region and in the areas around Rome, is compensated for by the high proportion of urban areas at national level. NK scores were in fact closely related to the degree of urbanization, age, education, and working status (53, 54).

Our data demonstrated that at population level, in Italy, the diet-disease association was the NK section with the highest scores (60.2%), while the lowest scores were found in the food composition section (53.5%). Different findings were reported in the assessment mentioned above (32) in which the highest NK rate was achieved in the Experts’ Recommendations section (59%) and the lowest in the association between diet and diseases (44%). Socio-economic aspects could partially explain these differences that may be related to the sampling methods and bias in the selection of the study group. The better knowledge of experts’ recommendations found in Scalvedi et al. (32) could be related to the fact that the sample included only parents of school-age children, and was focused more on knowledge of specific topics including which foods to avoid and which to promote, while the general population may have a higher awareness of a topic such as diet-disease association, which is commonly found in general communication channels and is the target of public health nutrition actions.

Low AMD in Italy was reported in several studies (17, 18, 55, 56) even though the assessments were carried out using different methodologies. In Grant et al. (33) AMD was assessed on a population sample following the same methodology used in this study; the results were very similar in terms of percentage of low and low to moderate AMD (62 vs. 63%). With this nationwide assessment we were able to confirm that in Italy AMD is significantly associated with sociodemographic characteristics among women, the elderly, people with high educational levels, and those living in urbanized areas showing the highest AMD. The association between socioeconomic status and diet quality is well documented in literature. In high-income countries, both adults and children with higher socioeconomic status tend to have healthier diets than those with lower socioeconomic status (57, 58). Although to different degrees, the correlation of AMD with sociodemographic characteristics is confirmed in other Italian studies. For example, Vitale et al. (59) reported higher adherence in the elderly, in people with highly qualified employment, and with higher income. Moreover, lifestyle habits such as interest in reading food labels and frequent physical activity are also associated with high AMD. Dinu et al. (60) in a random sample collected with the use of a web-based Medi-Lite questionnaire confirmed the highest scores in women, the elderly and, individuals with a university degree. On the other hand, a cross-sectional study carried out by Biasini et al. (61) did not confirm the associations of AMD with age and geographical area of residence. Such discrepancies in the various studies may reflect the lack of representation of subjects from the various sociodemographic indicators in the study sampling. The timing of data collection could also be a further external factor influencing the AMD. Even outside of the lockdown period - that in Italy was gradually reduced from the 18th of May 2020- the influence of the pandemic on dietary habits must be considered. The effect of COVID-19 impacted dietary practices both negatively and positively throughout Europe. Several studies reported an increase in the quality of the diet with increased consumption of healthy foods and increased AMD (62, 63). However, the higher quantity of food consumed during the restriction period was associated with other poor lifestyle outcomes, including weight gain and limited physical activity (64). Due to the specific objective of assessing the territorial variability and cultural diversity in Italy, the sampling of the present study was carried out so as to cover Italian macro-regions and the high-density population regions. Very interesting results were gained from this assessment, showing a well-defined North-South gradient both for NK and AMD.

Regions in the north-east and Campania (south) showed the lowest AMD (respectively, 45.4 and 44.2%), while Emilia-Romagna (north) and Lazio (central), as well as the islands (Sicily and Sardinia), showed the highest AMD (17.2, 16.2, and 17.7%, respectively). North-Central regions registered higher NK scores (Lazio, 29.5% and Emilia Romagna, 27.3%) than Southern regions (Molise 3.3%). According to de Silva et al. (65), further confirmed by Vilarnau et al. (16) south Mediterranean basin regions are progressively abandoning Mediterranean diet principles as a result of the globalization of food consumption patterns. The regional trend observed in this study is confirmed by the results of a cross-sectional investigation in South Italy that found a significant decrease in adherence to the MD from the 1980s to the 2000s, mainly in younger groups (18). On the other hand, in the adult population living in the north of Italy, no significant change in MD adherence from 1991 to 2006 was observed (66).

The data collected in this study showed that urbanization is a factor influencing NK and AMD, with the highest indexes found in areas with high population density. These findings are found also in other studies in which it was confirmed that, in many European countries, the diet of residents in rural areas differed from the principles of healthy eating habits compared to the diet of urban residents (67–69). False beliefs about nutrition could be caused by the unfavorable economic and social situation common in rural areas, often characterized by a low level of education, limited possibilities of getting a well-paid job, and reduced availability of healthy—often expensive—food (70, 71).

Analysis of the NK sections showed that in Italy there are several areas of food and nutrition literacy that need to be addressed. The knowledge of the different typologies of lipids (saturated, monounsaturated, polyunsaturated fatty acids, and cholesterol) and their presence in foods (butter, olive oil, sunflower oil, eggs) is limited. A further area that needs clarification is the recommendation for fruits and vegetable consumption that, as reported by Koch et al. (29), is not yet understood as a fundamental healthy choice. Our data showed consumers had difficulties in identifying the correct composition of certain foods, such as sweetened cola drinks considered high in sugar or breakfast cereals considered low in salt. More than half of the sample also stated that they were not aware of the meaning of classes of BMI. All these elements should be considered in the planning of educational campaigns.

The assessment of AMD revealed that a low proportion of the population achieved the recommended consumption frequencies of nuts (15.2%), fish (12.2%), legumes (10.1%), vegetables (8.9%), fruits (6.7%), and whole-grain cereals (8.8%). These aspects are partially confirmed by the national food consumption survey carried out in Italy in 2005/2006 (72). Differences were found related to fruit and vegetable consumption that, in Leclercq et al. (73), stood at 432 g/day an adequate level according to the WHO recommendation (74)—while in the present study the level recorded was insufficient for the majority of the population. The weekly consumption of meat was still too high and only 33.3% of the sample reported the exclusive consumption of white meat. However, Italians have reduced their consumption of red meat in the last 20 years, considering that red meat intake was 700 g/week in 2005/2007 (73). Anecdotical evidence of the reduction of red meat consumption was reported, especially by the private sector, with a decrease in the sales at market level being seen. One of the aims of the next food consumption survey, that is currently ongoing in Italy, is to verify consumers’ attitudes toward red meat consumption.

One new aspect of the NK methodology (37) was that of the comprehension of food labels. Our data demonstrated that the Italian consumers could understand the sense of the pictorial image (see question E12 in Supplementary Material A) identifying the product with the highest energy content among two labels proposed as models. Instead, the capacity to understand the sense of the list of ingredients was found to be limited. These aspects will need further research in Italy considering that often in the public health nutrition campaigns, as well as in the Italian Food-based Dietary Guidelines (38), the importance of reading labels to make healthy food choices is underlined. However, if the population does not have sufficient knowledge to do so, this recommendation risks being ineffective.

Our data demonstrated that exploring both the diets of consumers and their knowledge related to nutritional recommendations and food composition is a valuable approach for the definition of effective strategies to shift dietary behavior toward that of the recommendations. Specifically, the mechanism capable of explaining the association between adherence to the MD and the ability to understand nutritional issues can reasonably rely on the consumers’ awareness of their dietary behavior. Nevertheless, further research on the potential mechanisms involved in this association is needed.

The strength of this study is represented by the sampling methodology that gave the national representativeness of the Italian adult population in terms of gender, age, income, and education as well as to explore regional differences. Another important added value of this assessment is the use of a questionnaire that had already been tested in Italy. The questionnaire was specifically designed to collect information on AMD and NK as the main outcome of the study, in line with the pre-determined objectives. However, this kind of study has the general limitation related to self-reported answers that could affect the reliability of the responses. Furthermore, the eating habits assessed were based on the participants’ perception of food intake that may not reflect true intake. However, the large sample size and the confirmation of our results with other similar surveys support the reliability of the data collected. The same limitation could be considered regarding self-reported, rather than measured, weight and height. However, the use of self-reported anthropometric measurements in adults can be used at population level for ponderal index classification purposes (75). Despite the large set of indicators collected, some aspects of the lifestyle were not addressed, such as physical activity level or smoking habits. Another aspect that is missing is the issue of sustainability—a concept that is still vague for Italian consumers (76, 77)—that is not addressed in AMD and NK questionnaires. As reported by Castellini et al. (78), the COVID-19 pandemic may have been the turning point to raise consumer awareness concerning the close interconnection between human health and ecosystems, supporting the “One Health” perspective and improving a sustainable diet, all of which are aspects of growing importance that need to be included in further similar assessments.

## Conclusion

This large study showed that the AMD in Italy is generally low and that a health dietary pattern is closely linked to the literacy of the population in terms of NK. Socioeconomic aspects were strong determinants of both adherence to the Mediterranean dietary pattern as well as of NK. Globalization has led to drastic changes to the food system which has resulted in “nutrition transition,” whereby traditional diets shift to highly processed food products and foods that are high in saturated or trans fats, refined sugars, salt, low in fiber, and less nutrient-dense (79). This transition is happening also in Italy, where there is a progressive abandonment of Mediterranean dietary patterns, which is mainly affecting areas with low socioeconomic indicators. The close connection between NK and healthy dietary behaviors is an important aspect to take into consideration in terms of the development of educational campaigns. In the light of the findings of this study, the use of NK as an instrument for the evaluation of the effectiveness and impact of policy actions should be taken into consideration.

## Data Availability Statement

The raw data supporting the conclusions of this article will be made available by the authors, without undue reservation.

## Ethics Statement

Ethical review and approval was not required for the study on human participants in accordance with the local legislation and institutional requirements. The patients/participants provided their written informed consent to participate in this study.

## Author Contributions

VA and LR contributed equally to conceiving, writing, and reviewing the manuscript. LR was responsible of overall supervision, project administration, and funding acquisition. Both authors have read and agreed with the published version of the manuscript.

## Conflict of Interest

The authors declare that the research was conducted in the absence of any commercial or financial relationships that could be construed as a potential conflict of interest.

## Publisher’s Note

All claims expressed in this article are solely those of the authors and do not necessarily represent those of their affiliated organizations, or those of the publisher, the editors and the reviewers. Any product that may be evaluated in this article, or claim that may be made by its manufacturer, is not guaranteed or endorsed by the publisher.
