# Supplementary material for: Nutrition Knowledge as a Driver of Adherence to the Mediterranean Diet in Italy
Source: Front Nutr. 2022 Mar 21;9:804865. doi: 10.3389/fnut.2022.804865 (PMC8978558; doi:10.3389/fnut.2022.804865)
Supplement: Supplementary file 1 [file Data_Sheet_1.docx]

Supplementary Material

# Supplementary Data

**A. The I-NK questionnaire: Italian Nutrition Knowledge questionnaire**

List of the questionnaire items measuring i) Nutrition knowledge related to experts recommendations; ii) Food's composition; iii) Food choices and nutrition labels; iv) Diet-disease associations.

| ***Experts recommendations*** |  |
| --- | --- |
|  | **C1.** What do you think the experts recommend that people should be eating for the following foods? (*eating more; eating less; no recommendation; I don’t know*)  *vegetables; water; fruit; high fibre foods; salty foods, sugary foods; processed and preserved meat; fatty foods.*  **C2.** How many servings of fruit and vegetables a day do you think experts are advising people to eat? (*1; 2, 4; 5 or more; I don’t know*)  **C3.** What do you think the experts recommend that people should be consume among the following fats? (*eating more; eating less; I don’t know*)  *saturated fats; trans fats; unsaturated fats.*  **C4.** Which type of milk do experts advice people to eat (s*kimmed or semi-skimmed milk; whole milk; I don’t know; both; no one, not should be consume*)  **C5.** How many times a week do you think experts are advising people to eat fish? (*3-4 times a week; 1-2 times a week; everyday; I don’t know*)  **C6.** What is the tolerated limit of alcohol intake (alcoholic beverages) per day? (*1 drink for male and female; 2 drinks for male and female; 2 drinks for male and 1 drink for female; 3 drinks for male and 2 drinks for female; I don’t know)*  **C7*.*** How many times a week do you think experts are advising people to do breakfast? (*everyday; 4 times a week; I don’t know; 3 times a week)*  **C8**. Two glasses of fruit juice a day how many servings of fruits and vegetables correspond to? (*0; 1; 2, 3; I don’t know*)  **C9**. According to the Guidelines for a healthy Italian diet, how many carbohydrates should be taken daily compared to the total caloric intake? (*1/3; ¼; ½; I don’t know*) |
| ***Food's composition*** |  |
|  | **D1.** Do you think these food products are high or low in added sugar? (*high; low; I don’t know*)  *cola drinks with sweeteners; ice cream; tomato ketchup; melon; natural yoghurt.*  **D2.** Do you think these food products are high or low in salt? (*high; low; I don’t know*)  *soup, canned beans; bread; red meat; frozen vegetables; breakfast cereals.*  **D3.** Do you think these food products are high or low in fiber? (*high; low; I don’t know*)  *oat flakes; potatoes with peel; bananas; white rice; pasta; eggs.*  **D4.** Do you think these food products are high or low in protein? (*high; low; I don’t know*)  *beans; nuts; cheese; chicken meat; butter; fruits.*  **D5.** Do you think these food products are high or low in starch? (*high; low; I don’t know*)  *potatoes; pasta; bread; nuts; cheese.*  **D6.** What is the main fat contained in the following foods? (*polyunsaturated; saturated; monounsaturated, cholesterol; I don’t know)*  *butter; eggs; olive oil; sunflower oil.*  **D7.** Which of these food products mainly contain hydrogenated fats? (*biscuits, cakes and sweets; sunflower oil; fish; eggs; I don’t know)*  **D8.** The amount of calcium in a glass of whole milk compared to a glass of skimmed milk is? (*the same; higher; lower; I don’t know)*  **D9.** In your opinion, which one of the following has the most calories for the same weight? (*sugar; fats; carbohydrates/starch; fiber; I don’t know*)  **D10.** In your opinion, processed foods compared to those poorly processed contain: (*more calories; more fiber; low salt, I don’t know*) |
| ***Food choices and nutrition labels*** |  |
|  | **E1.** If a person wants to buy a low sugar yogurt, which would choose? (*natural yogurt; yogurt fruit 0% fat; fruit yogurt dessert; I don’t know)*  **E2.** If a person wants to eat a dish in a restaurant, which is the option with the lowest fat content? (*pumpkin cream; spelt and bean soup, risotto with mushroom; I don’t know*)  **E3.** What is the healthiest and most balanced choice for a meal in a restaurant? (*grilled chicken breast, with raw oil, vegetables and potatoes; burger with sauces and fries; fried fish and salad; I don’t know*)  **E4.** Which of these is the healthiest and most balanced picnic lunch? (*sandwich with tuna and tomatoes + fruit + skimmed yogurt + water;* s*andwich with omelette (2 eggs) + juice fruit + skimmed yogurt + water; sandwich with cooked ham and cheese + fruit snack + fruit juice + water; I don’t know*)  **E5.** Which of these desserts is the healthiest alternative? (*apple pie; ice cream; tart with jam of plum; cheesecake; I don’t know*)  **E6.** Which of these vegetable combinations in a salad would count a better variety of vitamins and antioxidants? (*broccoli, carrots, tomatoes; red peppers, tomatoes, lettuce; lettuce, green peppers, cabbage; I don’t know*)  **E7.** If a person wants to reduce the fat content in the diet, but does not want to avoid French fries, which of the following types is the best choice? (*thicker chips; less thick chips; wrinkled chips; I don’t know*)  **E8.** In your opinion, what would be added to flavor food without adding additional fat or salt? (*aromatic herbs; soy sauce; ketchup; I don’t know*)  **E9.** In your opinion, which of the following cooking methods requires the addition of fat? (*frying, steaming;* *grill; I don’t know*)  **E10.** On the product’s label, in what order are the ingredients placed? (*descending order; there is no rule; ascending order*; *I don’t know*)  **E11**. In your opinion, light foods are always good option because they are low in calories? (*I disagree; I agree; I don’t know*)  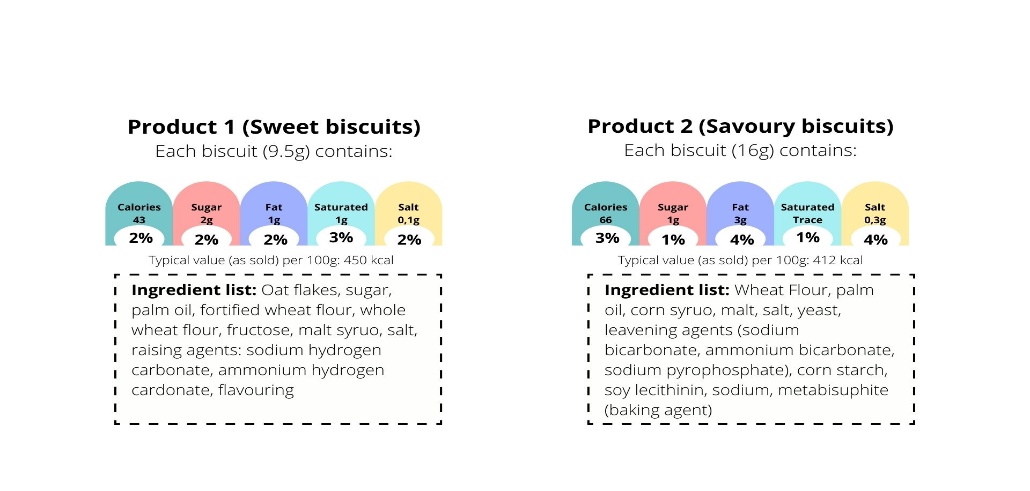  **Figure 1:** Nutrition labels used in the administration of questions.  **E12.** Considering the images of Product 1 and Product 2, which of the two has more calories (kcal) per 100 grams? (*product 1; product 2; they have same quantity; I don’t know*)  **E13**. What are the sources of sugar in the Product 1 ingredient list? (*sugar, fructose and malt syrup; sugar and malt syrup; sugar, fructose and lecithin; I don’t know*) |
| ***Diet-disease associations*** |  |
|  | **F1.** In your opinion, which of these diseases is related to low fiber content? (*intestinal disorder; anaemia; decay; I don’t know*)  **F2**. In your opinion, which of these diseases is related to sugar content in the diet? (*decay; hypertension; anaemia; I don’t know*)  **F3**. In your opinion, which of these diseases is related to salt content in the diet? (*hypertension; hypothyroidism; diabetes; I don’t know*)  **F4**. What do you think experts recommend to prevent cancer? (*eat less red meat; avoid additives in food; drink alcohol regularly; I don’t know*)  **F5**. What do you think experts recommend to people to prevent cardiovascular diseases? (*eat less trans fats; eat less fat fish; take supplements; I don’t know*)  **F6**. What do you think experts recommend to prevent diabetes? (*eat less refined foods; drink more fruit juices; eat more processed meat; I don’t know*)  **F7**. In your opinion, which of these foods is responsible for increasing blood cholesterol? (*animal fat; eggs; vegetable oils; I don’t know*)  **F8**. In your opinion, which of these foods has a high glycemic index? (*white bread, refined cereals; fruit and vegetables; I don’t know*)  **F9.** In your opinion, to maintain an optimal weight, people need to completely eliminate fat from the diet. (*I disagree; I agree; I don’t know*)  **F10**. In your opinion, to maintain an optimal weight, people need to follow a high-protein diet. (*I disagree; I agree; I don’t know*)  **F11**. In your opinion, eating bread always causes weight increase. (*I disagree; I agree; I don’t know*)  **F12**. In your opinion, eating fiber may decrease the risk of weight increase. (*I disagree; I agree; I don’t know*)  **F13**. In your opinion, which of these options can help people maintain an optimal weight? (*yes; no; I don’t know*)  *pay attention when eating; read nutritional labels; check the weight; no eat while watching tv; snack during the day; take supplements.*  **F14**. In your opinion, if a person has a Body Mass Index (BMI) of 23kg/m2, what is their nutritional status? (*normal weight; overweight; underweight; obese; I don’t know*)  **F15**. In your opinion, if a person has a Body Mass Index (BMI) of 31kg/m2, what will their nutritional status? (*normal weight; overweight; underweight; obese; I don’t know*)  **F16**. In your opinion, which of these body shape corresponds to an increase of cardiovascular risk? (*apple shape; pear shape; I don’t know*)  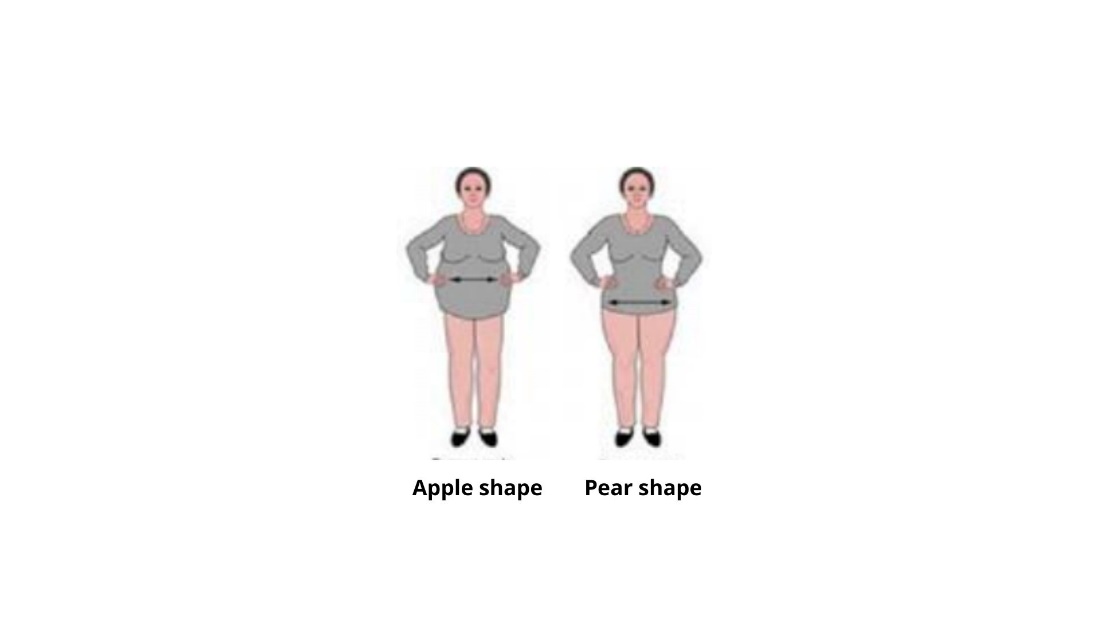  **Figure 2:** Body shapes. |

# 2.1 Supplementary Tables

**B. Table 3-Nutrition knowledge scores and sociodemographic characteristics**

| Table 3- Nutrition knowledge and sociodemographics | | | |  |  |  |  |  |  |  |  |  |  |  |  |  |
| --- | --- | --- | --- | --- | --- | --- | --- | --- | --- | --- | --- | --- | --- | --- | --- | --- |
| NK-General Nutrition Knowledge | **Total** | Gender | | Age (years) | | | | | | Education | | |  |  |  |  |
|  |  | Male | Fenale | 18-24 | 25-34 | 35-44 | 45-54 | 55-64 | > 64 | Low | Medium | High |  |  |  |  |
| Low (0-42) | **25.4%** | 29.1%_a_ | 22.0%_b_ | 28.3%_a,c_ | 36.3%_a,b_ | 42.7%_b_ | 19.7%_c,d_ | 17.5%_d_ | 17.8%_d,e_ | 31.2%_a_ | 22.6%_b_ | 13.3%_c_ |  |  |  |  |
| Low-Medium (43-52) | **26.5%** | 28.4%_a_ | 24.7%_b_ | 29.0%_a,b_ | 19.7%_a_ | 19.7%_a_ | 25.5%_a_ | 26.9%_a,b_ | 33.5%_b_ | 31.5%_a_ | 21.7%_b_ | 21.7%_b_ |  |  |  |  |
| Medium-High (53-60) | **26.0%** | 23.6%_a_ | 28.2%_b_ | 26.3%_a,b_ | 24.2%_a,b_ | 19.1%_a_ | 26.0%_a,b_ | 30.3%_b_ | 28.2%_b,c_ | 21.9%_a_ | 29.3%_b_ | 31.0%_b_ |  |  |  |  |
| High (>60) | **22.1%** | 18.9%_a_ | 25.1%_b_ | 16.4%_a_ | 19.9%_a_ | 18.5%_a_ | 28.8%_b_ | 25.2%_a,b_ | 20.6%_a_ | 15.4%_a_ | 26.4%_b_ | 34.0%_c_ |  |  |  |  |
|  |  |  |  |  |  |  |  |  |  |  |  |  |  |  |  |  |
| Table 3 (continued) |  |  |  |  |  |  |  |  |  |  |  |  |  |  |  |  |
| NK-General Nutrition Knowledge | **Total** | Region | | | | | | | | | | | | Degree of urbanisation | | |
|  |  | Alpin Regions (Piemonte, Liguria, Valle d'Aosta) | Lombardia | North east regions (Trentino Alto Adige, Friuli Venezia Giulia ) | Veneto | Emilia Romagna | Appennine regions (Tuscany, Umbria) | Adriatic regions (Marche, Abruzzo) | Lazio | Puglia and Molise | Campania | Calabria and Basilicata | Islands (Sardegna, Sicilia) | High | Medium | Low |
| Low (0-42) | **25.4%** | 22.2%_a,d,f,h_ | 30.3%_a,b,c_ | 39.5%_c_ | 26.8%_a,c,d_ | 16.1%_d_ | 17.5%_d,e,f_ | 32.0%_b,c,f,g_ | 17.6%_d,g_ | 23.7%_a,c,d_ | 31.6%_b,c,h_ | 22.1%_a,c,d_ | 27.9%_a,c,d_ | 20.2%_a_ | 27.1%_b_ | 39.2%_c_ |
| Low-Medium (43-52) | **26.5%** | 24.9%_a_ | 31.2%_a_ | 18.9%_a_ | 23.9%_a_ | 24.5%_a_ | 21.6%_a_ | 28.4%_a_ | 25.2%_a_ | 36.0%_a_ | 26.9%_a_ | 23.9%_a_ | 24.9%_a_ | 28.3%_a_ | 27.6%_a_ | 15.7%_b_ |
| Medium-High (53-60) | **26.0%** | 27.5%_a,d,e,f,g,i_ | 17.9%_a,b_ | 18.4%_a,c,d_ | 28.0%_a,d,e,f,g,i_ | 32.1%_d,e,f,g,i_ | 39.6%_e_ | 18.8%_b,c,f,h_ | 27.8%_a,d,e,f,g,i_ | 27.0%_a,d,e,f,g,i_ | 23.9%_b,c,g,h_ | 36.3%_d,e,h,i_ | 22.9%_b,c,i_ | 28.4%_a_ | 24.9%_a,b_ | 20.5%_b_ |
| High (>60) | **22.1%** | 25.4%_a,b_ | 20.6%_a,b_ | 23.2%_a,b_ | 21.3%_a,b_ | 27.3%_a_ | 21.3%_a,b_ | 20.8%_a,b_ | 29.5%_a_ | 13.3%_b_ | 17.6%_a,b_ | 17.7%_a,b_ | 24.3%_a,b_ | 23.1%_a_ | 20.4%_a_ | 24.6%_a_ |
|  |  |  |  |  |  |  |  |  |  |  |  |  |  |  |  |  |
| Table 3 (continued) |  |  |  |  |  |  |  |  |  |  |  |  |  |  |  |  |
| NK-General Nutrition Knowledge | **Total** | Household size (member number) | | | | | Household annual income | | | | | | |  |  |  |
|  |  | 1 | 2 | 3 | 4 | >=5 | <=18,000 € | 18,001- 27,000 € | 27,001- 36,000€ | 36,001 - 54,000€ | 54,001 -72,000 € | >=72,001 € | I prefer not to answer |  |  |  |
| Low (0-42) | **25.4%** | 24.8%_a_ | 17.7%_b_ | 30.3%_a,c_ | 27.5%_a_ | 39.7%_c_ | 28.1%_a_ | 25.0%_a_ | 26.0%_a_ | 24.7%_a_ | 24.1%_a_ | 24.1%_a_ | 29.9%_a_ |  |  |  |
| Low-Medium (43-52) | **26.5%** | 25.9%_a,c,d_ | 30.9%_a,b_ | 20.8%_c_ | 27.5%_b,d_ | 23.7%_a,c,d_ | 32.1%_a_ | 25.4%_a_ | 27.2%_a_ | 22.8%_a_ | 18.6%_a_ | 16.5%_a_ | 30.5%_a_ |  |  |  |
| Medium-High (53-60) | **26.0%** | 26.9%_a_ | 26.9%_a_ | 27.1%_a_ | 23.5%_a_ | 23.1%_a_ | 21.0%_a_ | 29.6%_b_ | 24.8%_a,b_ | 26.5%_a,b_ | 20.6%_a,b_ | 24.7%_a,b_ | 19.9%_a_ |  |  |  |
| High (>60) | **22.1%** | 22.4%_a,b_ | 24.5%_a_ | 21.9%_a,b_ | 21.4%_a,b_ | 13.5%_b_ | 18.8%_a_ | 19.9%_a,c_ | 22.0%_a,c_ | 25.9%_a,b_ | 36.7%_b_ | 34.8%_b,c,d_ | 19.7%_a,d_ |  |  |  |
| Values in the same row and sub-table that do not share the same index are significantly different at p< ,05 in the bilateral equality test for column proportions. | | | | | | | | |  |  |  |  |  |  |  |  |

**C. Table 4 - Adherence to Mediterranean Diet and sociodemographic characteristics**

| Table 4- Predimed Plus and sociodemographics | | | | |  |  |  |  |  |  |  |  |  |  |  |  |
| --- | --- | --- | --- | --- | --- | --- | --- | --- | --- | --- | --- | --- | --- | --- | --- | --- |
| Predimed Plus | **Total** | Gender | | Age (years) | | | | | | Education | | |  |  |  |  |
|  |  | Male | Fenale | 18-24 | 25-34 | 35-44 | 45-54 | 55-64 | > 64 | Low | Medium | High |  |  |  |  |
| Low (0<6) | **31.4%** | 36.6%_a_ | 26.6%_b_ | 39.9%_a_ | 36.6%_a,b_ | 35.2%_a,b_ | 31.4%_a,b,c_ | 27.6%_b,c_ | 26.4%_c_ | 35.0%_a_ | 29.5%_b_ | 24.6%_b_ |  |  |  |  |
| Low-medium (6-7) | **31.3%** | 31.7%_a_ | 30.9%_a_ | 34.8%_a_ | 26.9%_a_ | 35.9%_a_ | 31.8%_a_ | 32.4%_a_ | 28.6%_a_ | 31.6%_a_ | 30.5%_a_ | 32.3%_a_ |  |  |  |  |
| Medium-high (8-9) | **24.0%** | 22.0%_a_ | 26.0%_b_ | 18.0%_a_ | 23.9%_a_ | 20.7%_a_ | 26.3%_a_ | 25.8%_a_ | 25.3%_a_ | 22.1%_a_ | 26.0%_a_ | 25.7%_a_ |  |  |  |  |
| High (10-17) | **13.3%** | 9.7%_a_ | 16.6%_b_ | 7.3%_a_ | 12.6%_a_ | 8.2%_a_ | 10.6%_a_ | 14.2%_a,b_ | 19.8%_b_ | 11.4%_a_ | 14.1%_a,b_ | 17.4%_b_ |  |  |  |  |
|  |  |  |  |  |  |  |  |  |  |  |  |  |  |  |  |  |
|  |  |  |  |  |  |  |  |  |  |  |  |  |  |  |  |  |
| Table 4 (continued) | | | |  |  |  |  |  |  |  |  |  |  |  |  |  |
| Predimed Plus | **Total** | Region | | | | | | | | | | | | Degree of urbanisation | | |
|  |  | Alpin Regions (Piemonte, Liguria, Valle d'Aosta) | Lombardia | North east regions (Trentino Alto Adige, Friuli Venezia Giulia ) | Veneto | Emilia Romagna | Appennine regions (Tuscany, Umbria) | Adriatic regions (Marche, Abruzzo) | Lazio | Puglia and Molise | Campania | Calabria and Basilicata | Islands (Sardegna, Sicilia) | High | Medium | Low |
| Low (0<6) | **31.4%** | 30.1%_a,b_ | 32.8%_a,b,d_ | 45.4%_a,d_ | 34.4%_a,b,d_ | 27.0%_a,b_ | 26.3%_b_ | 30.7%_a,b,d_ | 34.0%_a,b,d_ | 23.6%_b,c_ | 44.2%_d_ | 24.2%_b,e_ | 25.0%_b,f_ | 31.4%_a_ | 30.6%_a_ | 34.3%_a_ |
| Low-medium (6-7) | **31.3%** | 32.6%_a,d,e_ | 33.0%_a,b,d,g_ | 18.6%_a,c,e,f_ | 27.8%_a,d,e_ | 33.3%_a,d,e_ | 40.6%_d_ | 29.8%_a,d,e_ | 20.8%_e_ | 36.4%_b,d,f,g_ | 23.2%_c,e,g,h_ | 39.1%_d,h_ | 37.0%_d,i_ | 27.7%_a_ | 34.0%_b_ | 35.5%_b_ |
| Medium-high (8-9) | **24.0%** | 22.4%_a_ | 24.0%_a_ | 24.6%_a_ | 26.0%_a_ | 22.4%_a_ | 19.5%_a_ | 27.8%_a_ | 29.0%_a_ | 30.3%_a_ | 22.7%_a_ | 21.7%_a_ | 20.2%_a_ | 26.2%_a_ | 22.3%_a_ | 21.9%_a_ |
| High (10-17) | **13.3%** | 14.9%_a_ | 10.2%_a_ | 11.4%_a_ | 11.7%_a_ | 17.2%_a_ | 13.6%_a_ | 11.7%_a_ | 16.2%_a_ | 9.8%_a_ | 9.9%_a_ | 15.0%_a_ | 17.7%_a_ | 14.7%_a_ | 13.1%_a_ | 8.3%_b_ |
|  |  |  |  |  |  |  |  |  |  |  |  |  |  |  |  |  |
| Table 4 (continued) | | | |  |  |  |  |  |  |  |  |  |  |  |  |  |
| Predimed Plus | **Total** | Household size (member number) | | | | | Household annual income | | | | | | |  |  |  |
|  |  | 1 | 2 | 3 | 4 | >=5 | <=18,000 € | 18,001- 27,000 € | 27,001- 36,000€ | 36,001 - 54,000€ | 54,001 -72,000 € | >=72,001 € | I prefer not to answer |  |  |  |
| Low (0<6) | **31.4%** | 22.5%_a_ | 28.2%_a,b_ | 32.2%_b,c_ | 37.4%_c_ | 42.2%_c,d_ | 29.8%_a_ | 29.7%_a_ | 28.0%_a_ | 32.1%_a_ | 37.2%_a_ | 34.3%_a_ | 33.1%_a_ |  |  |  |
| Low-medium (6-7) | **31.3%** | 30.6%_a_ | 29.9%_a_ | 33.8%_a_ | 31.6%_a_ | 29.6%_a_ | 32.9%_a_ | 35.5%_a_ | 28.6%_a_ | 27.7%_a_ | 22.5%_a_ | 34.6%_a_ | 29.9%_a_ |  |  |  |
| Medium-high (8-9) | **24.0%** | 33.1%_a_ | 24.7%_b_ | 21.5%_b_ | 21.9%_b_ | 19.9%_b_ | 26.9%_a_ | 23.7%_a_ | 28.4%_a_ | 22.7%_a_ | 23.3%_a_ | 18.6%_a_ | 20.5%_a_ |  |  |  |
| High (10-17) | **13.3%** | 13.8%_a,b_ | 17.2%_a_ | 12.5%_a,b_ | 9.1%_b_ | 8.3%_b,c_ | 10.3%_a_ | 11.1%_a_ | 15.0%_a_ | 17.4%_a_ | 16.9%_a_ | 12.5%_a_ | 16.4%_a_ |  |  |  |
| Values in the same row and sub-table that do not share the same index are significantly different at p< ,05 in the bilateral equality test for column proportions. | | | | | | | | | |  |  |  |  |  |  |  |
